# Influence of Graphene-Derivative Surface Chemistry on the Charge-Storage Behavior of Electrospun Cellulose Acetate Membranes

**DOI:** 10.3390/polym18151829

**Published:** 2026-07-26

**Authors:** Beatriz Reyes-Veloz, Liliana Licea-Jiménez, Sergio Alfonso Pérez-García

**Affiliations:** Centro de Investigación en Materiales Avanzados (CIMAV) Subsede Monterrey, Parque de Investigación e Innovación Tecnológica (PIIT), Av. Alianza Norte #202, Apodaca 66628, Nuevo León, Mexico; beatriz.reyes@cimav.edu.mx

**Keywords:** electrospinning, cellulose acetate, graphene derivatives, polymer nanocomposites, charge-storage mechanism, flexible electrodes, supercapacitors

## Abstract

Electrospun nanocomposite membranes have attracted considerable interests for flexible energy-storage devices; however, the influence of graphene derivative surface chemistry on the electrochemical behavior of cellulose acetate-based nanocomposites remains insufficiently understood. In this work, cellulose acetate (CA) nanocomposite membranes containing graphene oxide (GO), reduced graphene oxide (rGO), and octadecylamine-functionalized reduced graphene oxide (rGO-ODA) at concentrations of 0.1 and 0.2 wt% were fabricated by electrospinning and evaluated as electrode materials for supercapacitor applications. The interfacial interactions and morphology of the membranes were investigated by Fourier-transform infrared spectroscopy (FTIR) and scanning electron microscopy (SEM), respectively, while electrochemical performance was assessed by cyclic voltammetry in three-electrode and symmetric two-electrode configurations. FTIR analysis confirmed effective interactions between the graphene derivatives and the CA matrix, whereas SEM observations revealed that nanofiller chemistry influenced fiber morphology and structural homogeneity. Among the evaluated systems, GO-containing membranes exhibited the best electrochemical performance, reaching a specific capacitance of 5.982 F/g in the three-electrode configuration. Analysis of the charge-storage mechanism using the power-law relationship revealed distinct electrochemical behavior associated with the surface chemistry of each graphene derivative. The results demonstrate that graphene-derivative chemistry governs the structure-property relationships, electrochemical response, and charge-storage behavior of electrospun CA nanocomposites, providing fundamental insights for the design of lightweight and flexible electrodes for energy-storage applications.

## 1. Introduction

The growing demand for portable, lightweight, and flexible electronic devices, together with the global transition toward sustainable and renewable energy technologies, has intensified the demand for advanced energy storage systems with improved performance and multifunctionality. Among electrochemical energy storage technologies, supercapacitors have attracted considerable attention due to their high-power density, rapid charge–discharge capability, and long cycling stability often exceeding 100,000 cycles [1]. Despite these advantages, their energy density remains significantly lower than that of lithium-ion batteries, typically ranging between 10 to 10^3^ Wh/kg [2]. This limitation represents a major challenge in the development of next-generation, high-performance, and flexible power sources required for wearable electronics, hybrid energy systems, and electric vehicles.

Improving the electrochemical performance of supercapacitors depends strongly on electrode properties, as capacitance, power density, and long-term stability are governed by electrode composition, morphology, and interfacial structure [1,3,4]. Carbon-based nanomaterials, conducting polymers, and transition-metal oxides are among the most extensively investigated active components, each offering distinct advantages in energy or power density [5,6,7,8]. In this context, graphene and its derivatives, including graphene oxide (GO) and reduced graphene oxide (rGO), have emerged as particularly attractive because of their exceptional electrical conductivity, high theoretical specific surface area, and chemical stability [4,6]. Chemically modified graphene electrodes have demonstrated specific capacitances of approximately 135 F/g in aqueous electrolytes and 99 F/g in organic media [4,6,9]. However, these values remain below the theoretical capacitance estimated for ideal monolayer graphene. This performance gap is often caused by the restacking of graphene sheets driven by strong van der Waals interactions, which reduces the accessible surface area and restricts ion transport within the electrode. To address this issue, various strategies have been proposed, such as combining graphene derivatives with conducting polymers, metal oxides, or hierarchical carbon structures in order to prevent aggregation and create more efficient ion–electron pathways [5,8,10,11].

In parallel with advances in graphene-based electrodes, cellulose and its derivatives have attracted increasing attention as sustainable, low-cost, environmentally friendly structural polymers for flexible energy storage devices. Nanocellulose, as a cellulose-based composite, has demonstrated excellent mechanical properties, tunable surface chemistry, and the ability to host conductive reinforcements, enabling the development of lightweight and flexible architectures [12,13,14]. Early demonstrations of graphene–cellulose hybrid electrodes showed that vacuum-filtered graphene–cellulose papers can operate as flexible supercapacitor electrodes, achieving gravimetric capacitances on the order of 120 F/g, stable cycling, and excellent bending durability [15]. More recently, nanocellulose/graphene oxide hybrids, carbonized cellulose nanofibril/graphene aerogels, and cellulose-based three-dimensional scaffolds have been developed as free-standing, binder-free electrodes, delivering promising volumetric capacitance, energy density, and cycling stability for compact supercapacitors [14,16,17,18]. Comprehensive reviews highlight that nanocellulose/2D-material composites are among the most promising eco-friendly platforms for supercapacitor electrodes, but also emphasize that performance and scalability strongly depend on how graphene derivatives are dispersed and anchored within the cellulose network [13,14].

Within the family of cellulose derivatives, cellulose acetate (CA) is particularly attractive due to its abundance, tunable degree of substitution, solubility in relatively benign solvents, and excellent processability into films and fibers. Electrospun CA nanofibers have been explored in membranes and functional composites, including graphene oxide/CA nanofiber membranes for adsorption and separation applications and carbon black/CA nanofiber for oil-removal systems, demonstrating that CA can host conductive fillers in electrospun architectures with controlled morphology [19,20,21]. In the energy storage field, CA has also been combined with metal-organic frameworks and other conductive phases to produce flexible, free-standing electrodes for supercapacitors, showing that CA-based fibrous networks can provide mechanical integrity and porosity while accommodating electroactive components [22]. In addition, electrospun graphene oxide/cellulose acetate composite nanofibers have been mechanically characterized, revealing that low graphene oxide loadings can significantly enhance tensile strength and wettability, both of which are beneficial for electrochemical interfaces [21].

A key requirement for flexible supercapacitors is the development of binder-free, self-supporting electrodes in which the active material and current collector are integrated into a mechanically robust and electrically continuous architecture [11,16,18]. Conventional approaches, in which carbon powders are mixed with polymeric binders and cast onto metal foils, suffer from limited flexibility and additional interfacial resistance. Electrospinning provides a unique advantage in overcoming these limitations; it offers a high surface-to-volume ratio, interconnected porosity, and a tunable microstructure. These features facilitate ion diffusion, promote efficient electrolyte interaction, and enable the fabrication of free-standing, binder-free electrodes [19,23,24,25]. Multiple reviews converge on the conclusion that electrospinning-derived nanofibers, whether carbonized, polymer-based, or hybrid, represent one of the most promising pathways for next-generation flexible and wearable supercapacitors [23,24,25,26].

Despite significant progress in graphene-cellulose hybrid materials, the integration of electrospun cellulose acetate fibers, reinforced with graphene derivatives and specifically tailored as binder-free electrodes for supercapacitors, remains relatively underexplored. Most reported cellulose/graphene systems rely on paper-like structures, aerogels or nanocellulose films [14,15,16,17,18], while CA-based electrospun nanocomposites incorporating graphene derivatives have been studied mainly for adsorption, separation, or mechanical performance rather than for electrochemical energy storage [19,20,21,22]. Furthermore, although graphene oxide has been widely incorporated into polymer matrices, most previous studies have focused on enhancing electrochemical performance through changes in filler loading, conductive additives, carbonization treatments, or processing conditions, making it difficult to isolate the specific contribution of graphene-derivative surface chemistry. Consequently, systematic studies comparing different graphene derivatives (GO, rGO, and octadecylamine-functionalized reduced graphene oxide (rGO-ODA)) under identical experimental conditions to establish direct structure–property relationships governing nanofiber morphology, electrochemical response, and charge-storage behavior remain scarce.

Based on these considerations, this work focuses on the fabrication and electrochemical evaluation of electrospun cellulose acetate nanocomposite fibers incorporating graphene oxide (GO), reduced graphene oxide (rGO), and octadecylamine-functionalized reduced graphene oxide (rGO-ODA). Rather than simply demonstrating the beneficial effect of incorporating graphene derivatives into a polymer matrix, GO was intentionally selected as a reference material to enable a systematic comparison with rGO and rGO-ODA under identical reinforcement content and electrospinning conditions. This experimental design isolates the influence of graphene-derivative surface chemistry on polymer–nanoreinforcement interactions, fiber morphology, electrolyte accessibility, electrochemical response, and charge-storage behavior, providing a clearer understanding of the structure–property relationships governing electrospun cellulose acetate nanocomposite electrodes.

## 2. Materials and Methods

### 2.1. Synthesis of Graphene Derivatives

#### 2.1.1. Materials

Natural graphite flakes (lateral ≈ 5 μM) were used as the carbon precursor for the synthesis of graphene oxide (GO). Potassium nitrate (KNO_3_, 99.15%), potassium permanganate (KMnO_4_, 99.6%), and sulfuric acid (H_2_SO_4_, 96.35%) were purchased from CTR, Monterrey, México, and Fermont, Monterrey, México, respectively. Hydrogen peroxide (H_2_O_2_, 30%) and hydrochloric acid (HCl, 36.6%) were obtained from J.T. Baker, Phillipsburg, NJ, USA. For the preparation of reduced graphene oxide (rGO) and octadecylamine-functionalized reduced graphene oxide (rGO-ODA), ammonium hydroxide (NH_4_OH, Química Analytica, Monterrey, México), ascorbic acid-6-palmitate (99%, Mallinckrodt Chemicals, St. Louis, MO, USA), and octadecylamine (99%, Sigma-Aldrich, St. Louis, MO, USA) were used. All the chemicals were used as received without further purification.

#### 2.1.2. Synthesis of Graphene Oxide (GO)

Graphene oxide was synthesized from natural graphite flakes using the modified Hummers method [27]. Briefly, 2 g of graphite were dispersed in 120 mL of H_2_SO_4_ under continuous stirring. KNO_3_ (1.18 g) and KMnO_4_ (12 g) were gradually added while maintaining the reaction temperature below 20 °C to control oxidation. The reaction mixture was stirred at 450 rpm for 6 h. The reaction was terminated by carefully adding 400 mL of deionized water, followed by 24 mL of H_2_O_2_, which produced a color change indicating completion of oxidation. The dispersion was centrifuged at 3500 rpm for 10 min, and the supernatant was discarded. The solid product was washed with 10% *v*/*v* HCl and ionized water three times. After washing, 400 mL of deionized water were added, and the suspension was ultrasonicated for 1 h. Finally, the dispersion was centrifuged at 3500 rpm for 10 min, and the supernatant was collected. The final GO aqueous dispersion had a concentration of 5.033 mg/mL.

#### 2.1.3. Synthesis of Reduced Graphene Oxide (rGO)

The previously prepared GO dispersion was diluted to 1 mg/mL. An aqueous ascorbic acid solution (20 mg/mL) was prepared, and its pH was adjusted to 10 with ammonium hydroxide (NH_4_OH). The ascorbic acid solution was then added to the GO dispersion at 1:1 (*v*/*v*) ratio under continuous stirring at 450 rpm. The reduction reaction was allowed to proceed for 24 h at room temperature. After completion of the reaction, the suspension was centrifuged at 3500 rpm for 10 min, and the supernatant was discarded. The solid product was washed with 10% *v*/*v* HCl, followed by three washes with deionized water. The material was then redispersed in deionized water and ultrasonicated for 1 h. Finally, rGO was collected by vacuum filtration through a Teflon membrane and dried at 40 °C. The synthesis procedure followed the methodology described elsewhere [22].

#### 2.1.4. Synthesis of Octadecylamine-Functionalized Reduced Graphene Oxide (rGO-ODA)

An aliquot (134 mL) of the previously prepared GO aqueous dispersion was mixed with an ethanolic solution of octadecylamine (ODA) at a concentration of 0.01 g/mL of GO dispersion, using a 1:1 (*v*/*v*) ratio. The mixture was stirred to promote interaction between GO sheets and ODA. Subsequently, the system was phase transferred to toluene. Ascorbic acid-6-palmitate (680 μL of 10 μM solution) was added, and the reaction mixture was maintained under continuous stirring at 98 °C for 2 h at 450 rpm. Finally, the product was collected by vacuum filtration through a Teflon membrane, washed thoroughly with ethanol during filtration, and dried at 40 °C. The procedure followed the methodology reported elsewhere [23].

### 2.2. Preparation of Nanocomposite Materials and Electrospun Membranes

#### 2.2.1. Materials for Nanocomposites

Cellulose acetate (CA, average molecular weight of 30,000 g/mol, Sigma-Aldrich) was used as the polymer matrix for the fabrication of an electrospun membrane. Acetone (99.6%, J.T. Baker) and N,N-dimethylformamide (DMF, 99.8%, Alfa Aesar, Ward Hill, MA, USA) were used as solvents to prepare the polymer solutions.

#### 2.2.2. Preparation of Nanocomposite Electrospinning Solutions

Nanocomposite solutions were prepared using graphene derivatives (GO, rGO, and rGO-ODA) as reinforcing materials. Two filler contents were evaluated, corresponding to 0.1 and 0.2 wt% relative to the total solution volume. Cellulose acetate (CA) was dissolved at a concentration of 24 wt% in a solvent mixture consisting of N,N-dimethylformamide (DMF) and acetone in a 3:2 volume ratio. Separately, the required volume of the graphene derivative was ultrasonicated with a probe sonicator at 50% amplitude for 1 h to ensure homogeneous dispersion. The sonicated dispersion was then added to the CA solution and sonicated for 30 min at 50% amplitude. In total, six different formulations were prepared corresponding to the three graphene derivatives at two concentration levels.

To promote homogeneous nanofiller dispersion, each graphene-derivative solution was probe-sonicated for 1 h prior to mixing with the cellulose acetate solution, followed by an additional 30 min of probe sonication after polymer incorporation. Electrospinning was initiated immediately after dispersion preparation to minimize sedimentation or re-agglomeration before fiber formation. Although these procedures substantially improved dispersion homogeneity, localized agglomeration may still occur owing to the intrinsic tendency of graphene sheets to restack through van der Waals interactions, particularly after reduction.

Preliminary experiments were also performed using a graphene-derivative concentration of 0.5 wt%. However, these formulations exhibited poor electrospinning behavior due to increased solution viscosity and reduced dispersion stability, resulting in unstable jet formation and non-uniform fiber deposition. Therefore, concentrations of 0.1 and 0.2 wt% were selected because they provided stable electrospinning conditions while allowing the isolated evaluation of the influence of graphene-derivative surface chemistry on the electrochemical response.

#### 2.2.3. Fabrication of Electrospun Nanocomposite Membranes

Electrospun membranes were fabricated using a laboratory-scale electrospinning apparatus. The polymer nanocomposite solutions were loaded into a syringe fitted with a metal needle and electrospun at an applied voltage of 12 kV. The distance between the needle tip and the collector was fixed at 15 cm, and the solution feed rate was maintained at 1000 μL/h using a syringe pump. Two types of collectors were employed: aluminum foil and fluorine-doped tin oxide (FTO)-coated glass substrates. The electrospinning process was carried out under ambient laboratory conditions. After fabrication, the membranes were dried at room temperature to ensure solvent evaporation prior to further characterization and electrochemical testing.

To ensure that the effect of graphene-derivative surface chemistry could be evaluated independently, all electrospinning parameters, including applied voltage, solution flow rate, tip-to-collector distance, and deposition time, were maintained constant for every formulation throughout the study.

The electrospun membranes exhibited an average thickness of approximately 40 μm, determined from five independent measurements performed at different locations on a representative electrospun membrane. Since all formulations were prepared under identical deposition conditions, comparable membrane thicknesses were obtained, minimizing geometric effects on the electrochemical response.

#### 2.2.4. Characterization of the Electrospun Membranes

The surface morphology of the electrospun membranes was examined by scanning electron microscopy (SEM). The chemical interactions between cellulose acetate and the incorporated graphene derivatives were analyzed by Fourier-transform infrared spectroscopy (FTIR).

Electrochemical characterization was performed by cyclic voltammetry (CV) in two configurations. For the three-electrode configuration, electrospun membranes deposited on FTO substrates served as working electrodes. A silver/silver chloride (Ag/AgCl) electrode served as the reference electrode, and a platinum wire was employed as the counter electrode. Electrochemical measurements were performed using an aqueous electrolyte consisting of 1 M KNO_3_ containing 1 mM potassium ferricyanide (K_3_[Fe(CN)_6_]), where potassium ferricyanide was employed as a redox probe during cyclic voltammetry measurements.

For the two-electrode configuration, a symmetric capacitor-type assembly was constructed using electrospun membranes deposited on aluminum foil as both electrodes. The active electrode area was 1 cm^2^. A square filter paper separator (1.5 cm × 1.5 cm, pore size 90 μm) soaked in 1 mM potassium ferricyanide (K_3_[Fe(CN)_6_]) solution was used as both separator and electrolyte. Copper adhesive tape was used as an electrical contact.

The active mass corresponded exclusively to the electrospun membrane deposited on the substrate and was determined for each electrode prior to electrochemical testing. The mass loading ranged from approximately 1–2 mg for the three-electrode configuration and 3–4 mg for the symmetric two-electrode configuration. All gravimetric capacitance values reported in this work were normalized using the active mass of each individual electrode.

## 3. Results and Discussion

Prior to their incorporation into the cellulose acetate matrix, the graphene derivatives (GO, rGO, and rGO-ODA) were independently characterized by scanning electron microscopy (SEM), X-ray diffraction (XRD), Raman spectroscopy, and thermogravimetric analysis (TGA) to verify their morphology, structural organization, degree of reduction, functionalization, and thermal stability. The complementary characterization confirmed the expected structural and physicochemical characteristics of GO, rGO, and rGO-ODA before nanocomposite fabrication, providing confidence that the differences observed in the electrospun nanocomposite membranes arise from the controlled surface chemistry of the graphene derivatives rather than from unintended variations in the starting graphene derivatives. A summary of these physicochemical characteristics is presented in Table S1, while the corresponding characterization results are provided in the Supplementary Materials (Figures S2 and S3). These analyses establish the physicochemical basis for interpreting the interfacial interactions, morphology, and electrochemical behavior of the electrospun nanocomposite membranes discussed throughout this work.

### 3.1. Interfacial Interaction in Electrospun Nanocomposite Membranes

**Figure 1:**
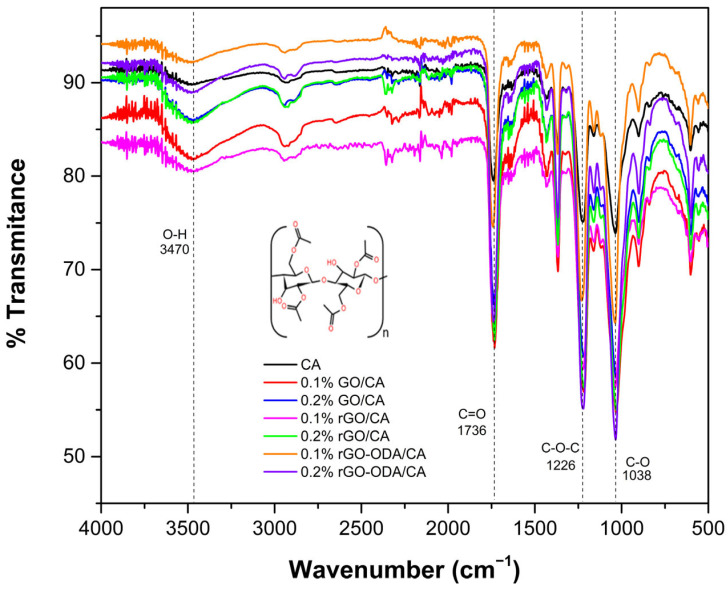
FTIR spectra of electrospun nanocomposite membranes based on cellulose acetate (CA) and graphene derivatives (GO, rGO, and rGO-ODA) at different concentrations (0.1 and 0.2 wt.%).

The chemical interactions between cellulose acetate (CA) and the incorporated graphene derivatives (GO, rGO, and rGO-ODA) were analyzed by FTIR spectroscopy using the electrospun membranes (Figure 1).

Considering the low graphene-derivative concentrations employed in this study (0.1 and 0.2 wt%), FTIR analysis was not intended to directly identify the characteristic functional groups of the graphene derivatives within the electrospun membranes. Instead, the analysis was used to evaluate changes in the vibrational response of the cellulose acetate matrix associated with interfacial interactions induced by the incorporation of graphene derivatives.

Pristine CA shows characteristic absorption bands related to its chemical structure. The range between approximately 1030 and 1100 cm^−1^ is due to C-O and C-O-C stretching vibrations from the polysaccharide backbone and acetyl groups, while the band near 1230 cm^−1^ corresponds to asymmetric C-O-C stretching of glycosidic linkages and acetate functionalities. A strong absorption band around 1730 cm^−1^ is linked to the stretching vibration of the ester carbonyl group (-O-C=O-CH_3_), a key feature of cellulose acetate. Additionally, a broad band centered around 3400 cm^−1^ is attributed to O-H stretching vibrations, from residual hydroxyl groups and hydrogen-bonded moisture within the polymer matrix. These features provide a baseline for assessing the impact of nanoreinforcement incorporation [28].

Incorporating graphene derivatives resulted in subtle yet consistent changes in the FTIR spectra. Specifically, band broadening in the hydroxyl region and minor variations in the carbonyl region indicate the formation of interfacial interactions between the polymer matrix and the nanoreinforcements. These modifications suggest hydrogen-bonding interactions between oxygen-containing functional groups in the graphene derivatives and the polar groups of CA. Therefore, the observed spectral variations should be interpreted as indirect evidence of polymer–nanoreinforcement interactions rather than as direct identification of graphene-derived functional groups within the electrospun membranes.

The extent of these interactions depends on the surface chemistry of each graphene derivative. GO, characterized by a high density of oxygenated functional groups, is expected to promote stronger polar interactions with CA, which is consistent with the more noticeable spectral changes observed in GO-containing membranes. In contrast, rGO, with a lower concentration of oxygen functionalities, exhibits comparatively weaker contributions in these regions. Meanwhile, rGO-ODA, due to the presence of long alkyl chains introduced during functionalization, is expected to enhance compatibility with the organic polymer matrix, potentially favoring improved dispersion during electrospinning.

Overall, the FTIR results confirm the successful formation of CA-based nanocomposite membranes with effective interfacial interaction between the polymer matrix and the graphene derivatives. These interactions are critical to ensuring homogeneous dispersion of nanoreinforcement during electrospinning and contribute to the structural integrity of the resulting fibers. Furthermore, the nature of these interactions is expected to play a key role in determining the electrochemical behavior of the membranes, particularly regarding charge storage mechanisms and ion accessibility within the fibrous network. The complete characterization of the graphene derivatives, including FTIR, SEM, Raman spectroscopy, XRD, and TGA, is provided in the Supplementary Materials (Figures S2 and S3 and Table S1), serving as the physicochemical basis for interpreting the behavior of the electrospun nanocomposite membranes presented in this study.

### 3.2. Morphology of the Nanocomposite Membranes

**Figure 2:**
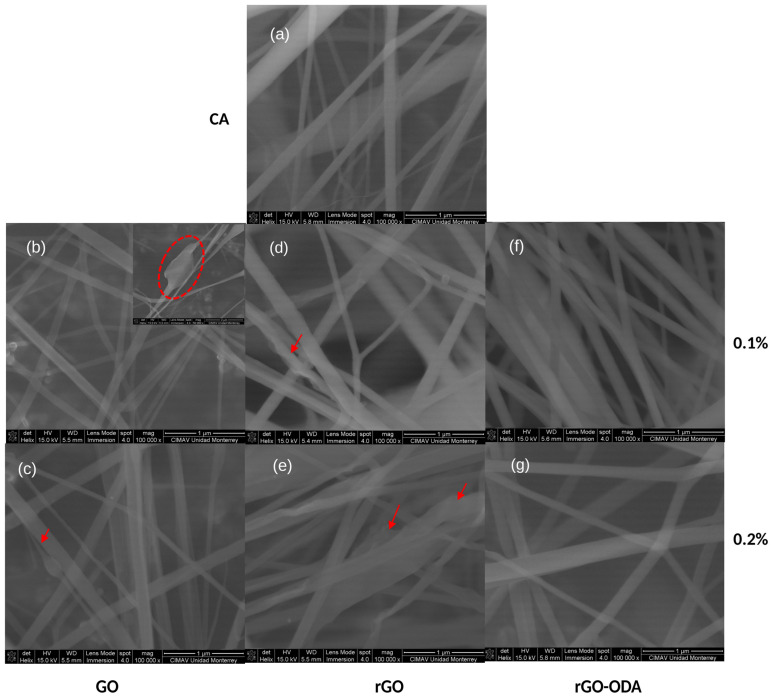
SEM micrographs of electrospun nanocomposite membranes at 100,000× magnification: (**a**) neat CA; (**b**) GO/CA (0.1 wt%); (**c**) GO/CA (0.2 wt%); (**d**) rGO/CA (0.1 wt%); (**e**) rGO/CA (0.2 wt%); (**f**) rGO-ODA/CA (0.1 wt%); and (**g**) rGO-ODA/CA (0.2 wt%). Localized morphological features consistent with nanoreinforcement-rich regions are indicated by arrows.

The morphological features of the electrospun membranes were examined using scanning electron microscopy (SEM), as shown in Figure 2. Furthermore, SEM observations were used to identify localized morphological features that are consistent with the incorporation of graphene derivatives. However, SEM does not directly resolve the spatial distribution of individual graphene sheets within the polymer matrix; therefore, the observed features are interpreted in conjunction with the complementary FTIR and electrochemical analyses.

The neat CA membrane displays a continuous, uniform fibrous network with smooth surfaces and relatively consistent fiber diameters. This morphology aligns with the well-known electrospinning behavior of cellulose acetate systems, in which appropriate solution viscosity and chain entanglement facilitate the formation of defect-free fibers [29].

The incorporation of graphene oxide (GO) into the CA matrix causes noticeable changes in fiber morphology. At both concentrations (0.1 and 0.2 wt%), the membranes containing GO show decreased uniformity, with localized irregularities along the fiber axis as shown in Figure 2b,c. These features can be linked to the abundance of oxygen-containing functional groups in GO, which promote strong hydrogen-bonding interactions with CA, as previously identified in the FTIR analysis (Section 3.1). While such interactions improve interfacial adhesion, they can also destabilize the electrospinning jet, resulting in bead formation and fiber deformation, a phenomenon commonly observed in polymer/GO systems [30,31].

In contrast, membranes incorporating reduced graphene oxide (rGO) exhibit a different morphological response. Although the fibrous network is preserved, the fibers show greater variability in diameter and occasional surface roughness. This behavior is associated with the lower content of oxygen functional groups in rGO, which weakens polymer–nanoreinforcement interactions and promotes partial agglomeration of the nanomaterial within the spinning solution. As a result, localized instabilities during jet elongation can occur, affecting fiber continuity and uniformity [31].

A significant improvement in morphological homogeneity is observed for membranes containing functionalized reduced graphene oxide (rGO-ODA). The presence of octadecylamine chains enhances compatibility between the nanoreinforcement and the CA matrix through hydrophobic interactions and steric stabilization. This improved dispersion leads to a more stable electrospinning process, resulting in fibers with smoother surfaces and a more consistent diameter distribution compared to GO- and rGO-based systems. Similar morphological improvements resulting from the surface functionalization of graphene derivatives have been reported in the literature, highlighting the importance of interfacial engineering in nanocomposite systems [32].

To understand the origin of localized morphological irregularities, representative high-magnification SEM micrographs highlighting bead-like structures and fiber distortions are provided in the Supplementary Materials (Figure S3). These localized morphological features are consistent with variations in the interfacial interactions between the cellulose acetate matrix and the different graphene derivatives. Although SEM alone cannot directly confirm nanofiller dispersion, the observations are consistent with the complementary FTIR and electrochemical results discussed in the following sections.

Overall, the observed morphological evolution—from uniform CA fibers to more irregular GO- and rGO-based systems, and finally to improved homogeneity in rGO-ODA composites—demonstrates the critical role of nanoreinforcement chemistry and surface functionality in governing fiber formation. These structural differences are directly correlated with the interfacial interactions identified by FTIR (Section 3.1) and are expected to influence the electrochemical behavior of the membranes, particularly ion transport pathways, effective surface area, and electrode/electrolyte interface characteristics [1].

### 3.3. Electrochemical Response in a Three-Electrode Configuration

**Figure 3:**
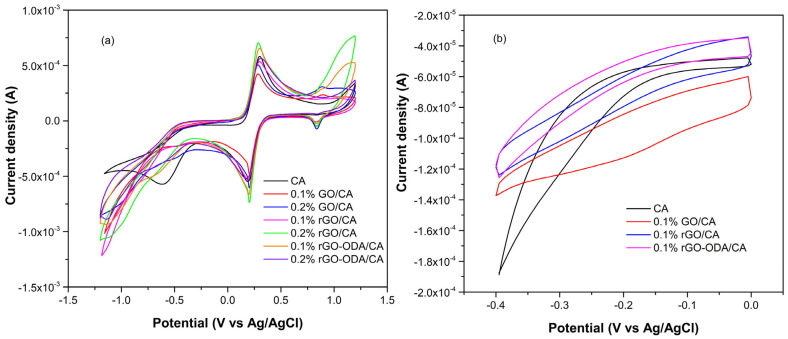
Electrochemical behavior of electrospun nanocomposite membranes in a three-electrode configuration. (**a**) Cyclic voltammetry (CV) curves of CA and nanocomposite membranes containing GO, rGO, and rGO-ODA at different concentrations (0.1 and 0.2 wt%) recorded at 100 mV s^−1^ within a potential window of −1.5 to 1.5 V. (**b**) CV curves of CA, 0.1% GO/CA, 0.1% rGO/CA, and 0.1% rGO-ODA/CA recorded at 50 mV s^−1^ within the capacitive potential window (−0.4 to 0 V).

The electrochemical behavior of electrospun cellulose acetate (CA) membranes and their corresponding nanocomposites incorporating graphene derivatives (GO, rGO, and rGO-ODA) was evaluated by cyclic voltammetry in a three-electrode configuration. The comparative CV profiles recorded at 100 mV s^−1^ within a wide potential window (−1.5 to 1.5 V) are presented in Figure 3a.

The pristine CA membrane exhibits a limited current response, consistent with its intrinsically insulating nature and low electroactive surface area. In contrast, all nanocomposite membranes exhibit higher current density, indicating enhanced charge storage capability upon incorporation of graphene-based nanoreinforcements. This improvement is commonly attributed to the formation of conductive pathways and the increase in accessible surface area provided by carbon nanostructures, which facilitate charge accumulation at the electrode–electrolyte interface [1,2,33].

Among the evaluated systems, GO-containing membranes exhibit the most pronounced capacitive behavior, as evidenced by the larger enclosed area under the CV curve. This response is associated with the high density of oxygen-containing functional groups (e.g., hydroxyl, epoxide, and carboxyl groups), which improve wettability and promote ion diffusion within the electrode structure [4,34,35,36]. These functional groups also contribute to pseudocapacitive effects, enhancing overall charge storage beyond purely electrostatic mechanisms [37]. In contrast, rGO-based membranes exhibit a reduced current response. Although the reduction process partially restores the conjugated sp^2^ carbon network and improves electrical conductivity, it also decreases the concentration of oxygenated functional groups, thereby reducing surface polarity and limiting ion accessibility [33,36]. This trade-off between conductivity and surface functionality is widely reported in graphene-based electrodes for supercapacitor applications.

These results indicate that the electrochemical response cannot be explained solely by the intrinsic electrical conductivity of the graphene derivatives. Although reduction increases the intrinsic electronic conductivity of rGO, the superior electrochemical performance of the GO-containing membranes demonstrates that charge-storage behavior is governed by the combined effects of surface chemistry, nanofiller dispersion, interfacial interactions, electrolyte accessibility, and the insulating nature of the cellulose acetate matrix. Therefore, the balance between electronic transport and ion accessibility, rather than intrinsic conductivity alone, determines the overall electrochemical response of the electrospun nanocomposite membranes.

The rGO-ODA nanocomposites present an intermediate behavior. While their capacitive response is lower than that of GO-based systems, their CV profiles are more stable and reproducible. This can be attributed to improved dispersion and interfacial compatibility between the nanoreinforcement and the polymer matrix, as previously observed in SEM analysis. Surface functionalization with octadecylamine enhances compatibility with the organic matrix, reducing agglomeration and yielding more homogeneous fiber structures, thereby favoring consistent electrochemical performance [31,38]. However, the presence of hydrophobic alkyl chains can partially hinder ion transport, resulting in a moderate electrochemical response compared to GO-based systems.

To further analyze the charge storage mechanism, representative CV curves were evaluated within a narrower potential window (−0.4 to 0 V) at reinforcement content of 0.1 wt%, as shown in Figure 3b. In this region, the curves exhibit a quasi-rectangular shape, characteristic of electric double-layer capacitance (EDLC) behavior, in which charge storage occurs via electrostatic adsorption of ions at the electrode surface [34,35]. Within this region, the 0.1 wt% GO/CA membrane shows the highest current response, confirming its superior capacitive performance. Meanwhile, the CA membrane maintains minimal activity, and the 0.1 wt% rGO-ODA/CA shows moderate behavior, reflecting the balance between improved structural integration and reduced surface functionality.

Overall, the results demonstrate that the electrochemical performance of the nanocomposite membranes is strongly governed by the interplay between surface chemistry and dispersion within the polymer matrix. The presence of oxygenated functional groups in GO enhances ion transport and charge storage, whereas functionalization strategies such as ODA modification improve structural homogeneity, highlighting the importance of interfacial engineering in the design of polymer-based supercapacitor electrodes.

A comprehensive set of electrochemical data, including cyclic voltammetry curves for all evaluated compositions and concentrations, is provided in the Supplementary Materials (Figures S4 and S5). These data support the trends discussed in Figure 3 and confirm the consistency of the electrochemical response across the different nanocomposite systems.

### 3.4. Electrochemical Response in a Symmetric Two-Electrode Configuration

**Figure 4:**
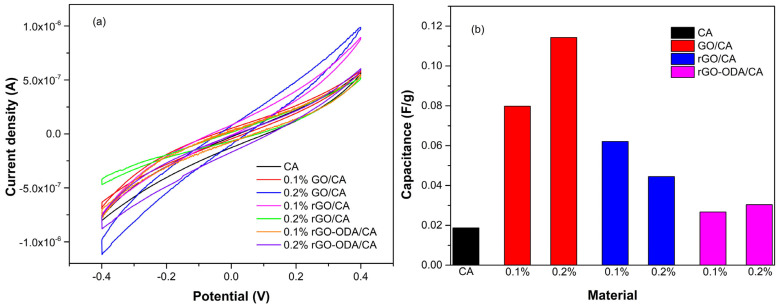
Electrochemical performance of electrospun nanocomposite membranes in a symmetric two-electrode configuration. (**a**) Cyclic voltammetry curves of CA and nanocomposite membranes containing GO, rGO, and rGO-ODA at different concentrations (0.1 and 0.2 wt%) recorded at 5 mV s^−1^ within a potential window of −0.4 to 0.4 V. (**b**) Gravimetric capacitance values calculated from the voltammetry curves for all evaluated membranes in the two-electrode configuration.

To evaluate the practical applicability of the electrospun membranes as supercapacitor electrodes, cyclic voltammetry was performed in a symmetric two-electrode configuration. Figure 4a compares the voltammetric response of CA and all nanocomposite membranes at a scan rate of 5 mV s^−1^. Under these conditions, the neat CA membrane exhibits the lowest current response, consistent with its limited electrical conductivity and low electroactive contribution.

After incorporating graphene derivatives, all nanocomposite systems exhibit an increase in the enclosed CV area, indicating improved charge storage capability. The voltammograms preserve an elongated quasi-rectangular shape, which is commonly associated with capacitive behavior in carbon-based electrode systems. This result confirms that the addition of graphene-derived nanofillers promotes a more favorable electrochemical response than the polymer matrix alone.

Among the evaluated materials, the GO/CA membranes exhibit the highest response, with 0.2% GO/CA showing the largest voltammetric area. This trend aligns with the capacitance values derived from the CV curves (Figure 4b), where the maximum gravimetric capacitance in the two-electrode configuration reaches 114.3 mFg^−1^ for the 0.2% GO/CA membrane. The improved performance of GO-containing systems can be attributed to oxygenated functional groups, which enhance electrolyte accessibility and facilitate charge storage at the electrode/electrolyte interface.

The rGO/CA membranes also exhibit improved capacitance relative to neat CA, although their performance remains below that of GO/CA. This behavior suggests that the partial reduction of oxygen-containing groups, while beneficial for electrical conductivity, decreases the number of electrochemically accessible surface sites. In the case of rGO-ODA/CA, the increase in capacitance is more modest, indicating that although surface functionalization improves compatibility and dispersion within the polymer matrix, the presence of alkyl chains may limit ion accessibility under device-like conditions.

Overall, the two-electrode results confirm the same qualitative trend observed in the three-electrode configuration: the incorporation of graphene derivatives improves the electrochemical performance of CA membranes, with GO providing the most significant enhancement. Importantly, the symmetric two-electrode arrangement provides a more realistic representation of device operation, supporting the feasibility of these electrospun nanocomposite membranes as lightweight, flexible electrode platforms.

The complete set of cyclic voltammetry curves obtained at different scan rates in the two-electrode configuration is provided in the Supplementary Materials (Figure S7), confirming the consistency of the trends discussed in the main text.

### 3.5. Charge-Storage Mechanism and Structure–Property Relationship

**Figure 5:**
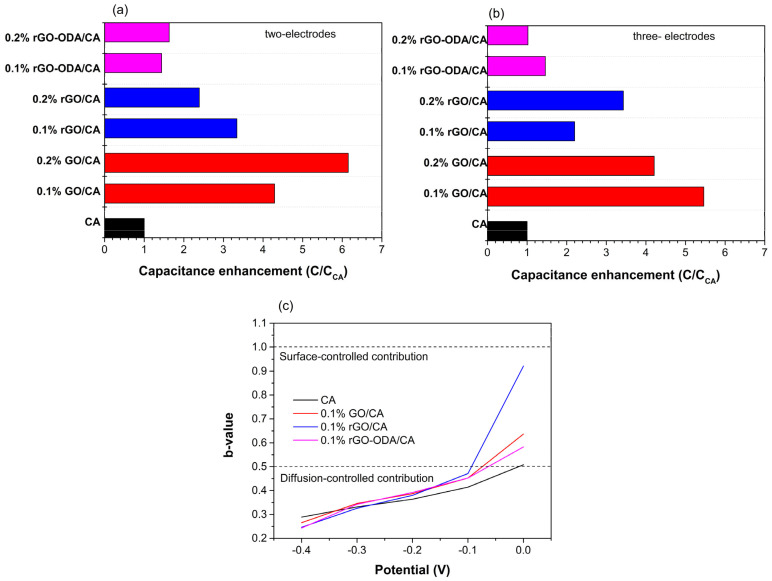
Capacitance enhancement values calculated from the ratio between the capacitance of nanocomposites and the capacitance of CA for all evaluated membranes in (**a**) the two and (**b**) three-electrode configurations. (**c**) Potential-dependent b-values for all electrospun nanocomposite membranes, including CA and membranes containing GO, rGO, and rGO-ODA at 0.1 wt%, calculated from scan-rate-dependent cyclic voltammetry measurements within the capacitive potential window.

To further understand the role of graphene-derivative chemistry in the electrochemical response of the electrospun membranes, the capacitance enhancement relative to neat CA and the charge-storage mechanism were analyzed as functions of composition and potential (Figure 5).

The capacitance enhancement factor shown in Figure 5a,b demonstrates that all nanocomposite membranes exhibit improved electrochemical response compared to pristine CA. However, the magnitude of the enhancement strongly depends on the chemical nature of the incorporated graphene derivative. In both electrochemical configurations, GO/CA membranes exhibit the greatest capacitance enhancement, indicating that oxygen-containing functional groups are critical for enhancing electrolyte accessibility and charge storage. The enhancement factor was calculated according to the relationship $C/C_{CA}$, where C corresponds to the gravimetric capacitance of the nanocomposite membrane and $C_{CA}$ represents the capacitance of the pristine cellulose acetate membrane under the same experimental conditions (Figure 5c). This behavior is consistent with previous reports describing the contribution of oxygen functionalities to pseudocapacitance and improved ion transport in graphene-based supercapacitors [37,38,39,40].

Although the intrinsic electrical conductivity of graphene derivatives increases after reduction, the electrochemical behavior of the electrospun nanocomposite membranes cannot be explained solely by this factor. Instead, the electrochemical response results from the combined influence of graphene-derivative surface chemistry, nanoreinforcement dispersion, polymer–nanoreinforcement interactions, fiber morphology, and electrolyte accessibility within the electrospun membrane.

The FTIR analysis discussed previously revealed modifications in the hydroxyl and carbonyl absorption regions after incorporation of graphene derivatives, particularly for GO-containing membranes, suggesting stronger interfacial interactions between the nanofillers and the CA matrix. These interactions are relevant because oxygen-containing groups such as hydroxyls, epoxides, and carboxyl functionalities improve wettability and facilitate electrolyte interactions with the electroactive surface [40]. 

The SEM analysis also supports the electrochemical trends observed in Figure 5a. GO-containing membranes preserved relatively continuous fibrous structures while maintaining accessible surface area, whereas rGO-containing systems exhibited greater morphological irregularities and partial fiber distortion. Although the incorporation of octadecylamine improved compatibility between rGO-ODA and the polymer matrix, leading to more homogeneous dispersion within the electrospun fibers, the presence of hydrophobic alkyl chains likely reduced electrolyte accessibility and limited the overall capacitance enhancement.

To gain insight into the charge-storage mechanism, the relationship between current response and scan rate was analyzed using the power-law equation:

$$i(V)=av^{b}$$

where i(V) is the current at a specific potential, v is the scan rate, and a and b are adjustable parameters [41]. The b-value was obtained from the slope of the log(i) versus log(v) relationship. Values close to b=1 indicate predominantly surface-controlled processes associated with electric double-layer formation, whereas values approaching b=0.5 are characteristic of diffusion-controlled charge-storage processes involving pseudocapacitive contributions [41,42].

The potential-dependent b-values presented in Figure 5c demonstrate that the charge-storage mechanism strongly depends on both the applied potential and the graphene derivative incorporated into the electrospun membrane. The pristine CA membrane exhibits relatively low b-values across most of the evaluated potential window, indicating limited surface-controlled contribution and restricted ion transport through the polymer matrix.

Among the evaluated systems, the 0.1% rGO/CA membrane exhibits the highest b-values at low potentials, approaching values close to unity. This behavior suggests a dominant surface-controlled contribution associated with the partially restored sp^2^ carbon network after reduction, which facilitates rapid charge transfer at the electrode surface [43]. However, the progressive decrease in b-value with increasing potential indicates that diffusion-controlled processes become increasingly relevant across the evaluated potential window. 

In contrast, GO/CA membranes exhibit intermediate but comparatively stable b-values, consistent with a mixed charge-storage mechanism involving both surface-controlled capacitive processes and diffusion-assisted ion transport. This behavior correlates with the superior capacitance enhancement observed in Figure 5a and suggests that oxygen-containing functional groups contribute not only to improved wettability, but also to the formation of electrochemically accessible active sites. Similar behavior has been reported in graphene oxide-based supercapacitor systems where controlled oxidation enhances ionic accessibility while preserving sufficient electrical conductivity [37,44,45].

The 0.1% rGO-ODA/CA membrane exhibits intermediate behavior between GO/CA and rGO/CA. Although functionalization with octadecylamine improves nanofiller dispersion and compatibility within the electrospun network, the lower b-values suggest that the hydrophobic nature of the alkyl chains partially limits ion accessibility and reduces the contribution of surface-controlled charge storage.

Overall, the combined FTIR, SEM, cyclic voltammetry, and potential-dependent *b*-value analyses consistently support the proposed structure–property relationship governing the electrochemical response of the electrospun nanocomposite membranes. FTIR demonstrates differences in polymer–nanoreinforcement interfacial interactions associated with graphene-derivative surface chemistry, while SEM reveals corresponding variations in fiber morphology. Together with the potential-dependent *b*-value analysis, these complementary experimental observations indicate that the electrochemical behavior is governed by the balance between surface functionality, conductive pathways, nanofiller dispersion, and electrolyte accessibility. Accordingly, the proposed charge-storage mechanism should be interpreted as a structure–property relationship supported by the experimental evidence presented in this work rather than as direct proof of the individual physicochemical processes occurring at the electrode/electrolyte interface.

Although the present study establishes clear correlations between graphene-derivative surface chemistry and the charge-storage behavior of electrospun nanocomposite membranes, additional electrochemical analyses, including galvanostatic charge–discharge measurements, electrochemical impedance spectroscopy, and long-term cycling stability tests, will be necessary to fully assess their device-level performance. Therefore, the present work provides a fundamental structure–property framework for the rational design and subsequent optimization of flexible electrospun electrodes for supercapacitor applications.

## 4. Conclusions

This work demonstrated the fabrication of electrospun cellulose acetate (CA) nanocomposite membranes incorporating graphene oxide (GO), reduced graphene oxide (rGO), and octadecylamine-functionalized reduced graphene oxide (rGO-ODA) as potential flexible electrode materials for supercapacitor applications. The results show that the electrochemical behavior of these membranes is strongly governed by the surface chemistry of the graphene derivative incorporated into the CA matrix.

FTIR analysis confirmed the formation of interfacial interactions between CA and the graphene derivatives, while SEM observations revealed that nanofiller chemistry influenced fiber morphology, dispersion, and structural homogeneity. GO-containing membranes exhibited the most favorable electrochemical response, reaching a maximum specific capacitance of 5.98 Fg^−1^ in the three-electrode configuration. This behavior was attributed to the oxygen-containing functional groups of GO, which enhance electrolyte accessibility and promote charge storage at the electrode/electrolyte interface.

The charge-storage analysis further demonstrated that the different graphene derivatives induce distinct electrochemical mechanisms. rGO-containing membranes showed a stronger surface-controlled contribution, associated with the partially restored sp^2^ carbon network, whereas GO-containing membranes exhibited a mixed charge-storage behavior related to both surface functionality and ion accessibility. In contrast, rGO-ODA improved nanofiller compatibility and morphological homogeneity, but the hydrophobic alkyl chains introduced during functionalization appeared to partially limit ion transport and electrochemical accessibility.

Overall, these findings demonstrate that graphene-derivative surface chemistry is a key parameter for controlling the structure–property relationships and charge-storage behavior of electrospun CA nanocomposite membranes. The results provide useful design criteria for developing lightweight, flexible, and binder-free polymer nanocomposite electrodes for future energy-storage devices.

## Data Availability

The original contributions presented in the study are included in the article/Supplementary Materials; further inquiries can be directed to the corresponding authors.

## Supplementary Materials

The following supporting information can be downloaded at: https://www.mdpi.com/article/10.3390/polym18151829/s1, Table S1: Characterization of graphene derivatives, as evidenced by exfoliation and the presence of oxygen-containing functional groups comparing with starting graphite. Figure S2: FTIR spectra of cellulose acetate (CA), graphene oxide (GO), reduced graphene oxide (rGO), and octadecylamine-functionalized reduced graphene oxide (rGO-ODA). Figure S3: High-magnification SEM images highlighting localized morphological irregularities in electrospun nanocomposite membranes. Figure S4: Cyclic voltammetry (CV) curves of electrospun nanocomposite membranes in a three-electrode configuration for all compositions and concentrations evaluated, recorded at 100 mV s^−1^ within a potential window of −1.5 to 1.5 V. Figure S5: Cyclic voltammetry (CV) curves of nanocomposite membranes in the capacitive potential window (−0.4 to 0 V) for all compositions and concentrations evaluated. Figure S6: Cyclic voltammetry (CV) curves recorded at different scan rates for selected nanocomposite membranes in a three-electrode configuration. Figure S7: Cyclic voltammetry curves recorded at different scan rates for CA and all nanocomposite membranes in a symmetric two-electrode configuration. Figure S8: Potential-dependent b-values for all electrospun nanocomposite membranes, including CA and membranes containing GO, rGO, and rGO-ODA at 0.1 and 0.2 wt%, calculated from scan-rate-dependent cyclic voltammetry measurements within the capacitive potential window.

## Abbreviations

The following abbreviations are used in this manuscript:

CA	cellulose acetate
GO	graphene oxide
rGO	reduced graphene oxide
rGO-ODA	octadecylamine-functionalized reduced graphene oxide
