# Supplementary material for: Influence of Graphene-Derivative Surface Chemistry on the Charge-Storage Behavior of Electrospun Cellulose Acetate Membranes
Source: Polymers (Basel). 2026 Jul 26;18(15):1829. doi: 10.3390/polym18151829 (PMC13467792; doi:10.3390/polym18151829)
Supplement: Supplementary file 1 [file polymers-18-01829-s001.zip › polymers-4429859-supplementary.pdf]

Influence of Graphene-Derivative Surface Chemistry on the Charge-Storage Behavior of Electrospun Cellulose Acetate Nanocomposite Membranes

Beatriz Reyes-Veloz, Liliana Licea-Jiménez*, Sergio Alfonso Pérez-García*

Centro de Investigación en Materiales Avanzados (CIMAV) Subsede Monterrey, Parque de Investigación e Innovación Tecnológica (PIIT), Av. Alianza Norte #202, Apodaca 66628, Nuevo León, Mexico; beatriz.reyes@cimav.edu.mx

* Correspondence: liliana.licea@cimav.edu.mx (L.L.-J.); alfonso.perez@cimav.edu.mx (S.A.P.-G.)

T1. Physicochemical characterization of graphene derivatives used for electrospun nanocomposite fabrication

The graphene derivatives (GO, rGO, and rGO-ODA) were comprehensively characterized prior to nanocomposite fabrication using complementary physicochemical techniques. These analyses verified the successful oxidation, reduction, and functionalization processes and provide the physicochemical basis for interpreting the morphology and electrochemical behavior of the electrospun nanocomposite membranes discussed in the main manuscript.

The graphene derivatives used in this study were characterised by Scanning Electronic Microscopy (SEM), X-Ray Diffraction (XRD), Thermogravimetric Analysis TGA and Raman Spectroscopy prior to their incorporation into the cellulose acetate matrix (Table S1) such testing was performed to understand the principal chemical and morphology characteristics of synthesised nanomaterials.

SEM micrographs reveal that the morphology of the synthesized materials differs from that of the starting graphite, exhibiting wrinkled structures due to the incorporation of oxygen-containing functional groups into the graphitic lattice. Additionally, increased translucency, indicative of material exfoliation, was observed. XRD analysis confirmed these findings, showing a decrease in intensity, peak broadening, and shift of the characteristic (002) peak, indicating the formation of few-layer structures (≤ 5 layers).

Furthermore, the incorporation of functional groups is corroborated by TGA and Raman spectroscopy. TGA shows mass losses in the 100–360 °C range, corresponding to the decomposition of oxygen-containing groups, as well as a decrease in the maximum decomposition temperature (T_{max}) compared to graphite. Similarly, Raman spectroscopy reveals an increase in the defect-related peak (D band), resulting in a higher ID/IG ratio, indicative of increased structural disorder typical in graphene oxide materials.

Table S1. SEM, XRD, TGA and RAMAN characterization of graphene derivatives, as evidenced by exfoliation and the presence of oxygen-containing functional groups compared with starting graphite material.

Material	SEM	XRD	No. layers
Graphite	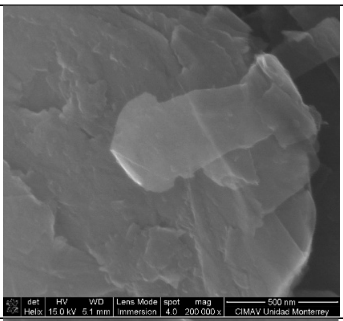	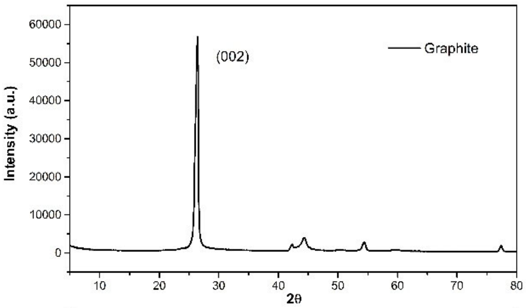	**43**
GO	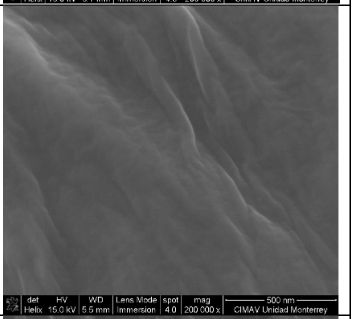	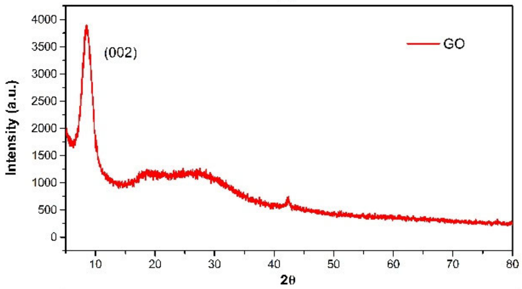	**5**
rGO	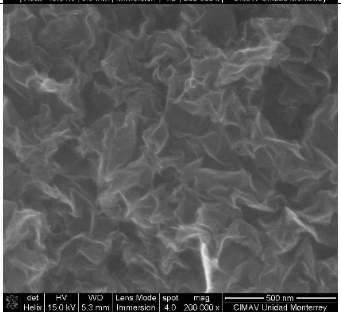	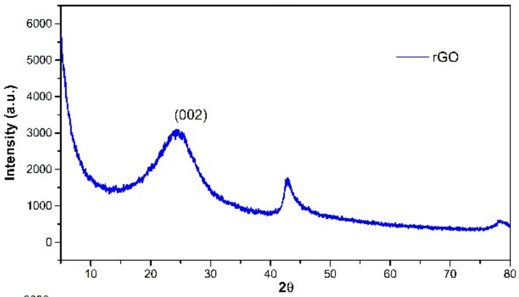	**3**
rGO-ODA	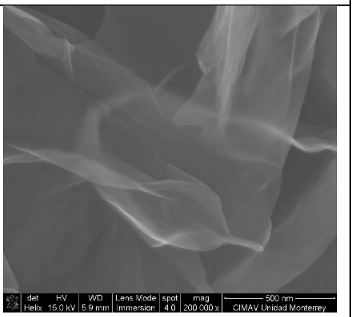	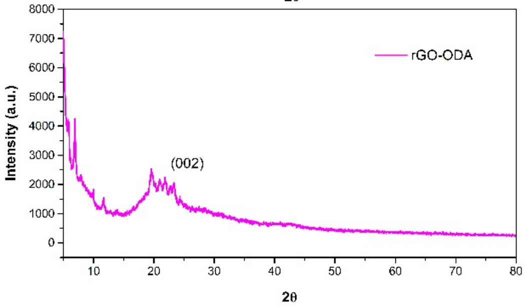	**4**

Material	TGA	T_{max}	Raman	Ration iD/iG
Graphite	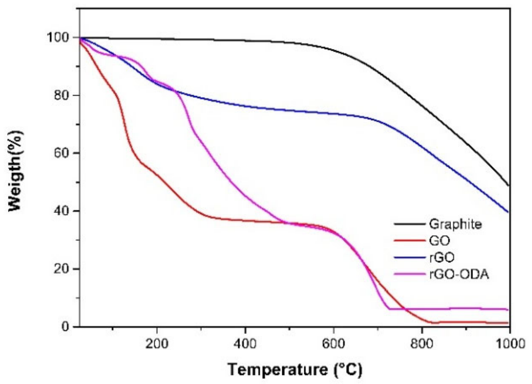	985	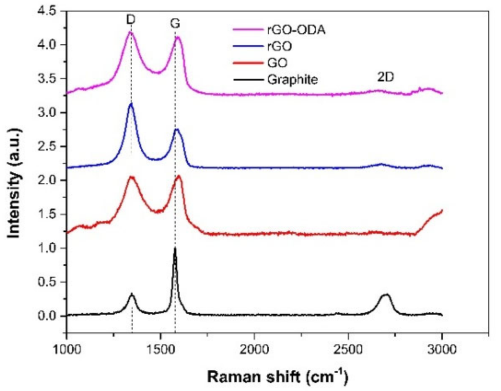	0.53
GO		662		1.33
rGO		826		1.57
rGO-ODA		700		1.19

T2. FTIR Characterization of Graphene Derivatives

The chemical structures of the graphene derivatives used in this study were analyzed by Fourier transform infrared spectroscopy (FTIR) prior to their incorporation into the cellulose acetate matrix (Figure S2).

Graphene oxide (GO) exhibited characteristic absorption bands associated with oxygen-containing functional groups, including a broad band around 3400 cm^{-1} corresponding to hydroxyl groups, a strong band near 1720 cm^{-1} assigned to carbonyl (C=O) stretching vibrations, and bands in the 1000–1300 cm^{-1} region associated with C–O functionalities. These features confirm the successful oxidation of graphite.

After chemical reduction, reduced graphene oxide (rGO) showed a noticeable decrease in the intensity of oxygen-related bands, indicating partial removal of oxygen functionalities and partial restoration of the graphitic structure.

In the case of rGO functionalized with octadecylamine (rGO-ODA), additional absorption bands were observed in the region of 2920 and 2850 cm^{-1}, corresponding to aliphatic C–H stretching vibrations. These features confirm the successful grafting of alkyl chains onto the graphene surface.

These differences in surface chemistry among GO, rGO, and rGO-ODA are expected to influence their interaction with the cellulose acetate matrix, as well as their dispersion behavior during electrospinning, ultimately affecting the structural and electrochemical properties of the resulting nanocomposite membranes.

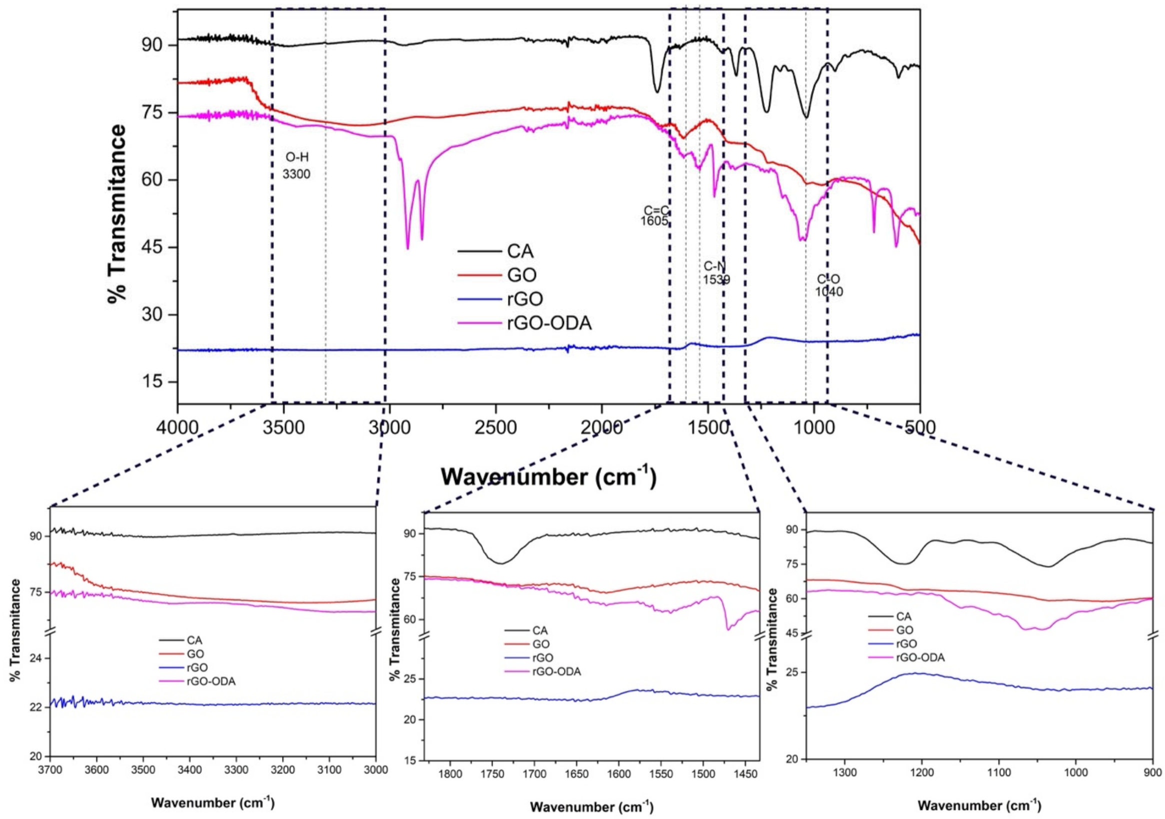

Figure S2. FTIR spectra of pristine cellulose acetate (CA), graphene oxide (GO), reduced graphene oxide (rGO), and octadecylamine-functionalized reduced graphene oxide (rGO-ODA). Enlarged views of the O–H, C=O, and C–O spectral regions are included to facilitate visualization of the spectral variations discussed in Section 3.1.

T3. Morphological irregularities in electrospun nanocomposite membranes

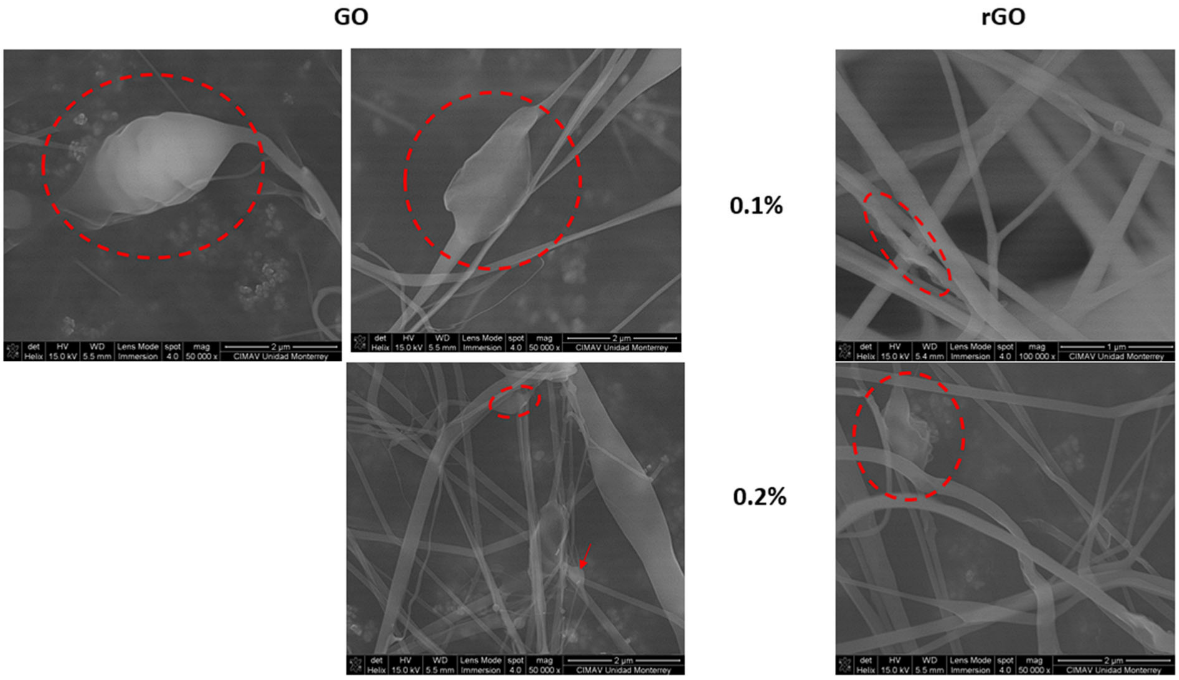

Figure S3. Higher-magnification SEM micrographs of representative electrospun nanocomposite membranes. The images provide additional morphological detail but are not intended to directly demonstrate the spatial distribution of graphene derivatives within the polymer fibers.

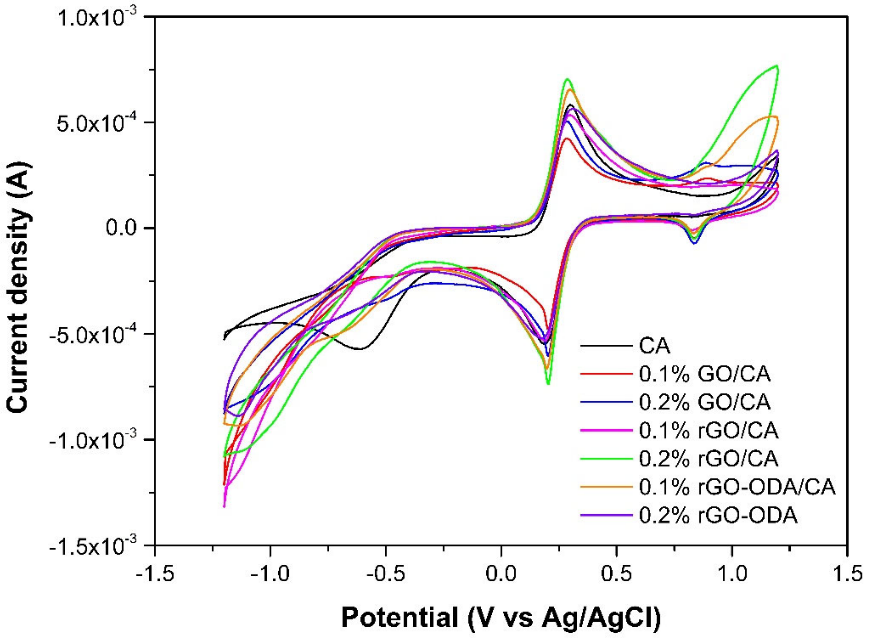

Figure S4. Cyclic voltammetry (CV) curves of electrospun nanocomposite membranes in a three-electrode configuration for all compositions and concentrations evaluated, recorded at 100 mV s^{-1} within a potential window of −1.5 to 1.5 V.

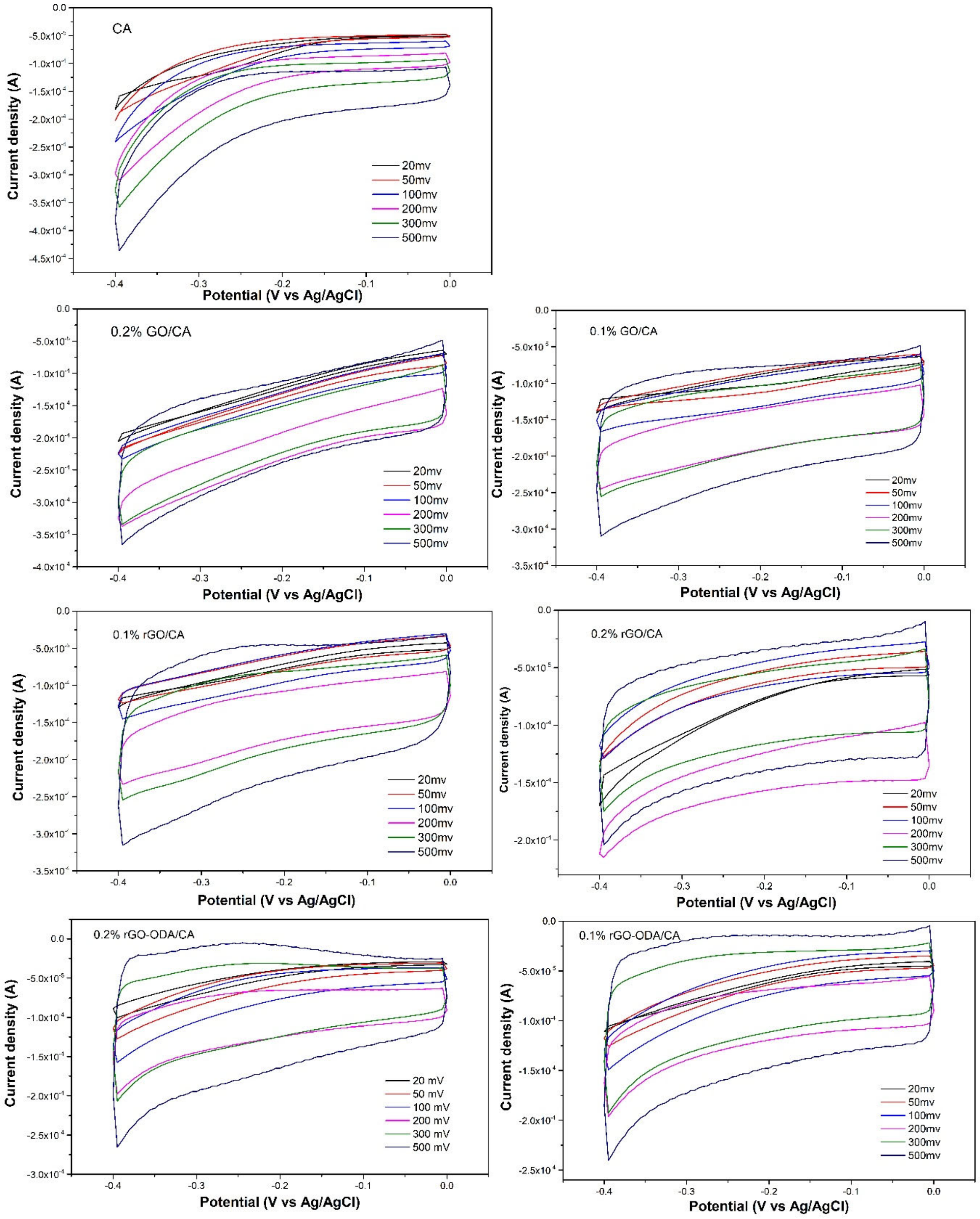

Figure S5. Cyclic voltammetry (CV) curves of nanocomposite membranes in the capacitive potential window (−0.4 to 0 V) for all compositions and concentrations evaluated.

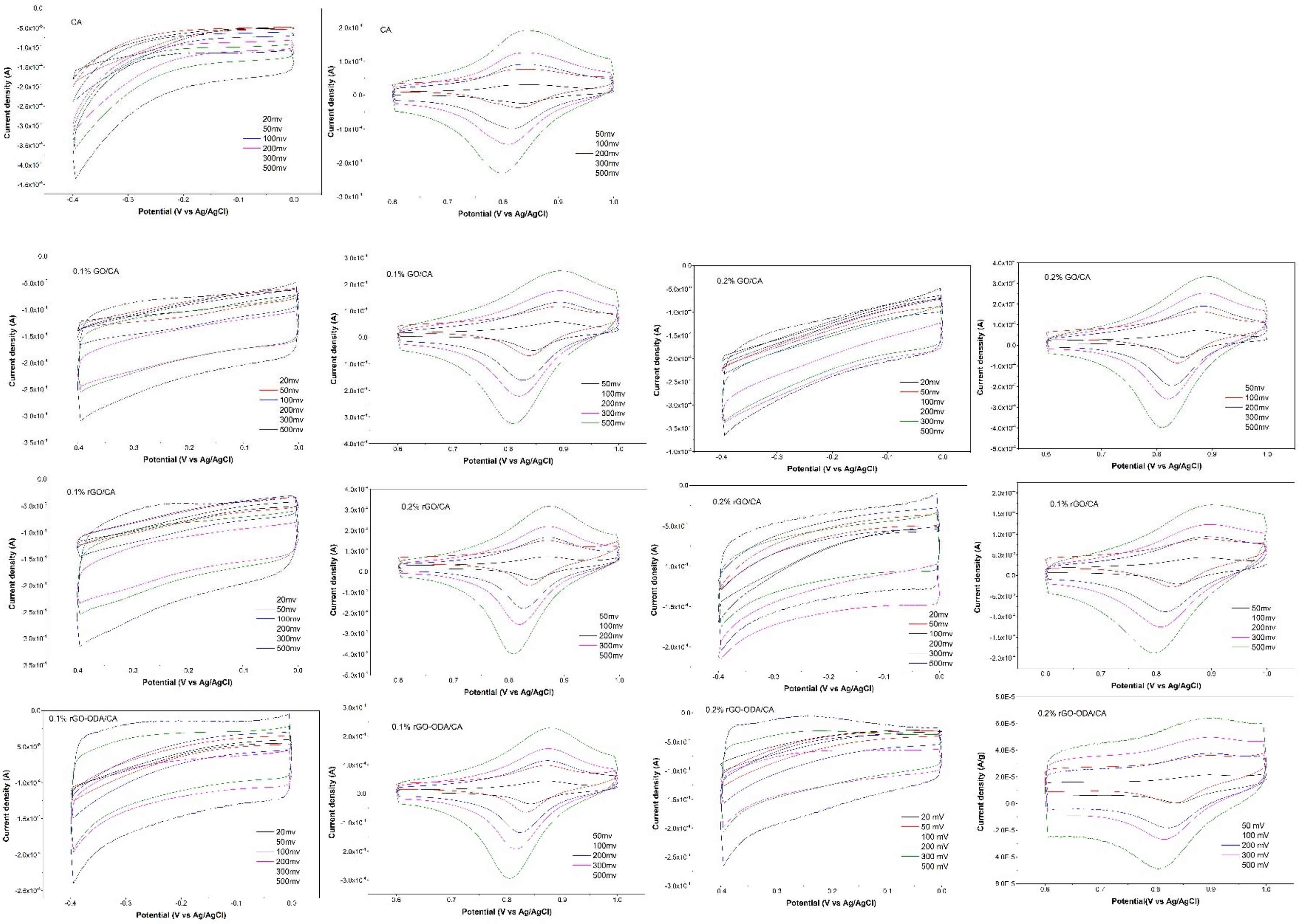

Figure S6. Cyclic voltammetry (CV) curves recorded at different scan rates for selected nanocomposite membranes in a three-electrode configuration.

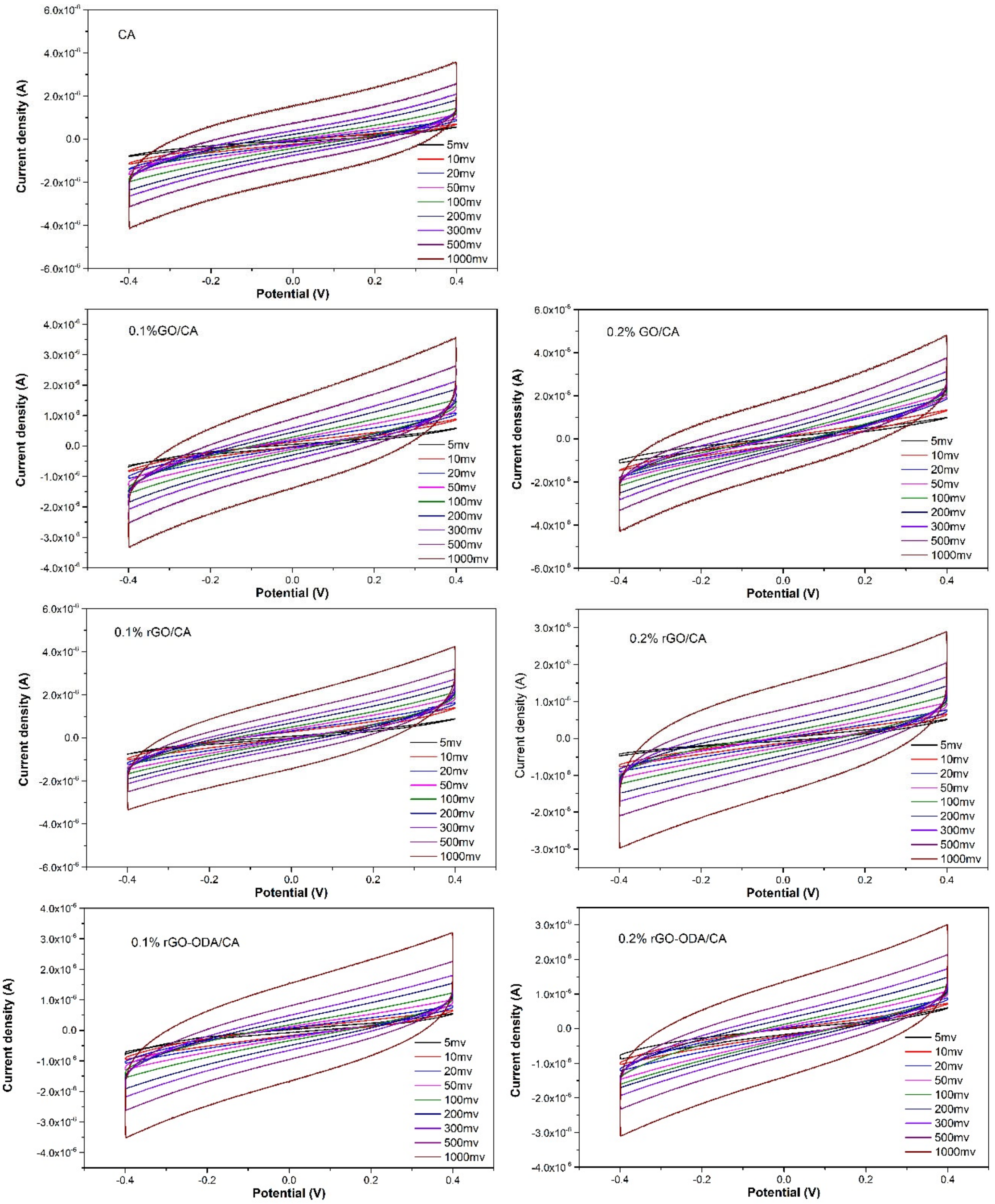

Figure S7. Cyclic voltammetry curves recorded at different scan rates for CA and all nanocomposite membranes in a symmetric two-electrode configuration.

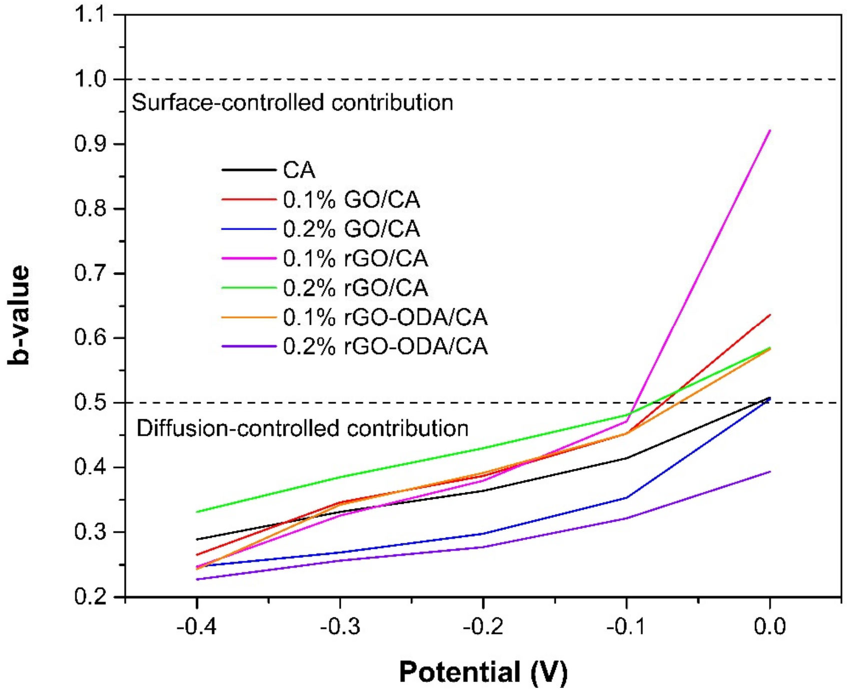

Figure S8. Potential-dependent *b*-values for all electrospun nanocomposite membranes, including CA and membranes containing GO, rGO, and rGO-ODA at 0.1 and 0.2 wt%, calculated from scan-rate-dependent cyclic voltammetry measurements within the capacitive potential window
